# Factors contributing to primary caregiver delay in presenting children with chronic kidney disease for medical care in Ghana

**DOI:** 10.4102/phcfm.v11i1.1894

**Published:** 2019-08-19

**Authors:** Abigail Amoah, Sinegugu E. Duma

**Affiliations:** 1 School of Nursing and Public Health, College of Health Sciences, University of KwaZulu-Natal, Durban, South Africa; 2 College of Health Sciences, University of KwaZulu-Natal, Durban, South Africa

**Keywords:** chronic kidney disease, childhood kidney diseases, medical care, primary caregivers, primary prevention

## Abstract

**Background:**

Conforming to the 2016 World Kidney Day focus on raising awareness of the early detection of kidney diseases in children, we report on factors that contribute to primary caregiver delay in presenting their children with chronic kidney disease (CKD) for medical care in Kumasi, Ghana.

**Aim:**

The objective of the study was to explore and describe the factors that contribute to primary caregiver delay in presenting children with CKD for medical care in Kumasi, Ghana.

**Setting:**

The study was conducted in the Paediatric Renal Unit in Kumasi, Ghana.

**Methods:**

A qualitative study was conducted in January 2017. Semi-structured interviews were used to collect data from a convenience sample of 10 primary caregivers whose children were admitted for CKD, but were not too ill. The primary caregivers had to respond to the research question: What factors contribute to your delay in presenting your child with CKD for medical care? Thematic data analysis and the ecological model of Schneider (2017) were used to organise the findings.

**Results:**

Four themes and related subthemes, including intrapersonal-related factors, interpersonal-related factors, community-related factors and infrastructural factors were identified as those that contribute to delay in presenting children with CKD for medical care.

**Conclusion:**

The findings show that primary prevention strategies for CKD in children should not only focus on personal-related factors but also cut across all levels of the socio-ecological model in order for them to be effective.

## Background

The theme of the 2016 World Kidney Day was ‘Kidney disease and children. Act early to prevent it’.^1^ This theme highlighted awareness about the relationship between childhood kidney disease and its progression to chronic kidney disease (CKD) in later life.^2^ It is therefore important for all primary health care practitioners to be cognisant of factors that may contribute to delayed medical action towards the prevention of the progression of acute childhood kidney disease into chronic disease. Primary health care practitioners should know the risk factors that may cause primary caregivers to delay in presenting their children for early detection and appropriate treatment of childhood kidney diseases in their communities. A qualitative study was chosen as the method to bring more insight to this issue.

Chronic kidney disease is the universal term for heterogeneous disorders affecting the kidney’s structure and function.^3^ It is a clinical term that is used when kidney damage reaches an irreversible state or where there is a reduction of kidney function which persists for 3 months or longer, with or without a decreased glomerular filtration rate.^4,5,6^

Early detection and treatment of kidney disease in children can help prevent complications and slow down its progress for both children and adults.^1,7,8,9,10,11^ Identifying the risk factors for CKD in children is important for the development of appropriate interventions to prevent the progression of kidney disease into adulthood.^1^

Unlike some parts of Africa, Ghana has limited access to chronic dialysis and kidney transplantation for children with CKD.^12^ This necessitates early detection and treatment of any kidney disease in children in order to reduce complications and progression to kidney failure.^7,9^ Despite evidence that early detection and treatment reduces complications and progression to kidney failure, it was observed that primary caregivers delayed presenting children with CKD for medical care to the Paediatric Renal Unit of a hospital in Kumasi, Ghana. Children were brought to the Paediatric Renal Unit with severe complications which were difficult to treat successfully, thus resulting in unnecessary deaths. This highlighted the need to explore and describe the primary caregivers’ perspectives on the factors that contribute to the delay in presenting children with CKD for medical care. Having insight into these perspectives on the factors contributing to this delay could then be used to inform the development of culturally appropriate and contextual strategies for early detection and treatment of kidney disease to prevent its progression.

## Method

A qualitative research design was used to explore and describe the factors that contribute to primary caregivers delaying the presentation of children with CKD for medical care. The research question was: What factors contribute to primary caregiver delay in presenting children with CKD for medical care in Ghana? Data were collected in January 2017.

### Study setting

The study was conducted in the Paediatric Renal Unit in Kumasi, Ghana. The hospital serves as a training facility for sub-speciality training for doctors and nurses, medical students, nursing students and other health professionals and serves most parts of Ghana and some neighbouring countries. The hospital records indicate that a total of 704 children with CKD were seen and treated between 2010 and 2014 in the Paediatric Renal Unit.^13^ In 2015 and 2016, 204 and 252 cases were reviewed, respectively, thus indicating an increase in the occurrence of CKD in the region.^13^

### Study population and sampling strategy

The study population were all primary caregivers, whose children were admitted to the Paediatric Renal Unit in Kumasi, Ghana. The nurse in charge of the Paediatric Renal Unit assisted in identifying suitable potential participants for recruitment. Convenient sampling of the caregivers who had delayed in presenting their children for medical care was performed. Those who agreed to participate in the study were then recruited individually and requested to participate in the study by signing the informed consent, thus indicating their willingness to voluntarily participate in the study. All participants were assured of their anonymity and informed of their rights as participants, including the right to withdraw from the study.

### Data collection

A pilot study was conducted with two participants in December 2016 in order to determine the suitability of the semi-structured interview guide to yield relevant data in accordance with the purpose of the study. Data from the pilot study were included in the analysis of the final results. This is common in qualitative studies where similar data collection and analysis methods are used in both the pilot and the main study.^14^

One-on-one interviews were conducted in Twi (the vernacular of the Ashanti Region), using a semi-structured interview guide and probing questions, which were developed by the authors to comply with the objective of the study. Each interview was audio recorded, and field notes were taken to capture the non-verbal communication of participants such as facial expressions and changes in sitting positions in response to interview questions. Field notes provided validation of relevant points made by participants and also facilitated appropriate emphasis on emergent themes.^15^ Each interview was transcribed within 24 hours. A professional language translator translated the transcribed interviews from Twi to English. This was further checked against the transcribed data by the authors to ensure that no information was lost in the translation process. Preliminary data analysis was done parallel to data collection in order to enhance verification of data, initiate member checking and determine data saturation.

### Data management and data analysis

All transcribed interviews were stored electronically in the researcher’s computer and backed up in Dropbox, using Microsoft Word documents, with each interview labelled with participant pseudonyms. All raw data, including field notes, were stored in a safe box and secured by the researcher with a pin-coded lock.

Thematic data analysis, as described by Polit and Beck,^16^ was used. The identified themes and subthemes were further organised according to the ecological model of Schneider^17^ as a framework that guided the study. Each transcript was read several times to obtain its feel and an overall sense of the content. Significant statements were identified from all transcripts using different coloured pens. The identified colour-coded significant statements aided to organise, identify, retrieve and analyse meanings from the data. Meaning was then formulated from the colour-coded significant statements to guide the analysis and then linked with the data to ensure that each significant statement was marked correctly. All formulated meanings were grouped into categories that reflected a unique structure of clusters of themes, using the ecological model of Schneider.^17^ The grouped clusters of themes that reflected significant statements were incorporated together to form a distinctive theme. Four main clusters of themes with subthemes extracted from the data were then identified as findings, which described the factors that contribute to primary caregiver delay in reporting children with CKD for medical care. Member checking was performed with all participants to validate the credibility of the identified themes.^16^ Other mechanisms to ensure trustworthiness of data included audit trail, peer debriefing and supervision of data analysis by an experienced qualitative researcher.

### Ethical considerations

Ethical clearance and permission to conduct the study were obtained from relevant authorities, including the University of Cape Town’s Human Research Ethics Committee (Ref: 402/2016) and the Ministry of Health in Ghana, prior to conducting the study.

## Findings

Eight participants identified themselves as mothers, while the other two identified themselves as fathers of the children with CKD. Only one female and one male participant reported to have obtained tertiary education certificates and were formally employed. The rest were illiterate and held different informal and menial jobs in their communities, including subsistence farming and being street vendors. This reflects the level of education in local communities in the northern sector of Ghana, where girls’ enrolment in schools remains lower than that of male learners.^18^ It also reflects how the poor socio-economic status in Ghana has pushed many of her citizens into farming, in an effort to earn money for their daily upkeep.^19^

Table 1 summarises the four themes and related subthemes as findings.

**TABLE 1:** Summary of themes and subthemes.

Themes	Subthemes
Intrapersonal-related factors contributing to delay in presenting children with CKD for medical care	Beliefs in traditional medicine as a cure for CKD Lack of knowledge about CKD Financial constraints Beliefs in ancestral and spiritual powers for healing
Interpersonal-related factors contributing to delay in presenting children with CKD for medical care	Parents’ marital conflict Lack of extended family support
Community-related factors contributing to delay in presenting children with CKD for medical care	Delayed referral from local health facility Mismanagement of disease at the local health facility Incorrect advice from neighbours
Infrastructural related factors	Poor roads Lack of transport

CKD, chronic kidney disease.

### Intrapersonal-related factors contributing to delay in presenting children with chronic kidney disease for medical care

This theme emerged from the data in relation to the individual caregiver’s personal characteristics, attitudes or abilities contributing to the delay in presenting their children for medical care. These were further grouped into four subthemes. Extracts from individual participants, using pseudonyms, were used as evidence to support the development of each subtheme.

#### Beliefs in traditional medicine as a cure for chronic kidney disease

The following extracts from the data show how the primary caregivers’ entrenched beliefs in the ability of traditional medicine to cure CKD contributed to the delay in seeking help for their children. This can be seen in the following extract from Afe (a female participant):

‘For two years, I made him drink traditional medicine from our family traditional healer. I also applied some of it on his navel regularly. I decided to seek medical care when his swelling was getting worse. I thought the traditional medicine was now failing to cure him.’ (Afe, female, 38 years)

Sheh (a male participant), in the following extract, further confirmed how beliefs in traditional medicine contributed to the delay in presenting his child to hospital:

‘My daughter drank the traditional medicine like tea every morning and evening for more than a year, but it could not cure her condition. Only then did I decide to bring her to the hospital.’ (Sheh, male, 55 years).

#### Lack of knowledge about chronic kidney disease

This subtheme was developed from the data indicating how the primary caregivers’ lack of knowledge about the symptoms of CKD contributed to delay in presenting their children for medical care, as demonstrated by the extract from Ade and Afe (two female participants) below:

‘Perhaps if I had insight about how this disease develops and progresses, I would have brought him to the hospital right away [rather] than to send him to the prayer camp.’ (Adeh, female, 40 years)

Another participant, Afe (a female participant) highlighted how lack of knowledge of the disease delayed her from seeking help for her child, given as follows:

‘I had never heard about the disease that does not heal like this. It was only when, until we were admitted here, that I learnt that this thing progresses badly. I would not have waited for three years if I had known.’ (Afe, female, 38 years)

#### Financial constraints

Primary caregivers’ lack of money emerged as a major subtheme on the intrapersonal level factors that contribute to delays in presenting a child for medical care. Hence, this subtheme was named ‘financial constraints’. The following extract from Afe (a female participant) affirmed this:

‘We didn’t have money so we tried traditional medicines because they are cheap, but none was able to cure him. Even the money to bring him here, we borrowed it because the swelling would not go away.’ (Afe, female, 38 years)

An extract from Abe, (a female participant) highlighted how financial constraints contributed to the delay in presenting her child with CKD for medical care:

‘It took me more than two weeks to make enough money from the market in order to return him for blood results. I didn’t have money. Perhaps if I [had] had it, they would have helped him.’ (Abe, female, 46 years)

Adam (a male participant) confirmed financial constraints as a contributory factor to his delay in seeking medical care for his child in the following extract:

‘It took me one year to gather enough money to finally bring him to XXX [name of hospital], where he had been referred more than two years ago.’ (Adam, male, 40 years)

#### Beliefs in ancestral and spiritual powers for healing

Participants’ beliefs in ancestral and spiritual powers were identified as another subtheme describing the factors that contribute to the delay in seeking medical care for their children, as demonstrated in the extracts from Ahe (a female participant) and Ateh (a male participant):

‘We first thought that both my husband and my own ancestors had conspired with my family ancestors to inflict the disease on our child because of his swelling. We sent him to a prayer camp where prayers were offered for him for two months, but his condition did not improve before we decided to bring him to the hospital.’ (Ahe, female, 44 years)

An extract from participant supports the subtheme as follows:

‘Our pastor said it was a spiritual problem. My wife stayed at the prayer camp with the child for two months for intensive prayers and anointing for our pastor to cast out the disease spiritually. When they came back, I saw that the child was not better and I brought him to the hospital, as his condition was worsening.’ (Adam, male, 40 years)

### Interpersonal-related factors contributing to delay in presenting children with chronic kidney disease for medical care

The two subthemes under this theme emerged from the data that described the influence of families, friends and neighbours as a contributory factor to the primary caregivers’ delay in seeking medical care for their children suffering from CKD, as can be seen from the extract below from two female participants:

#### Parental marital conflict

Parental marital conflict, including men refusing to listen to their wives’ requests or advice to take the children to hospital until it is too late, was identified as a contributory factor to the delay in seeking medical care for children, as can be observed in the following extracts from two women, Axe and Apeh, respectively:

‘My husband doesn’t listen to me when I talk about our children’s wellbeing. I begged him to allow me to bring the child to the hospital, but he refused, although he could see that the child was ill. It was only when [the] child’s condition deteriorated and he collapsed. Then we sent him to the hospital.’ (Axe, female, 42 years)

‘In my tribe I cannot challenge my husband’s word or decision. He chose his friends’ advice over mine and administered traditional medicine to our child for two months, which could not cure her. Eventually, when she had developed difficulty in breathing, I rushed her to the hospital without my husband’s consent.’ (Apeh, female, 48 years)

#### Lack of family support

Lack of family support was also described as a contributory factor that delayed seeking medical care for children with CKD, as demonstrated in the following extracts from the data from two female participants:

‘My husband is dead and nobody in my family was willing to help me, because they thought my child was going to die just like his father [crying]. I had to work on someone’s farm for six weeks for enough money to send my child to the hospital.’ (Ate, female, 47 years)

‘We did not get any support from my husband’s family. They blame my husband for marrying me, a disabled person [she has deformed legs].’ (Axe, female, 42 years)

### Community-related factors contributing to delay in presenting children with chronic kidney disease for medical care

The factors beyond the primary caregivers’ control were identified and described as three subthemes of community-related factors contributing to the delay in presenting children with CKD for medical car. These include delayed referral from the local health facility, mismanagement from the local facility and ill advice given by neighbours.

#### Delayed referral from the local health facility

This subtheme is captured in the following extracts from Axe and Adeh:

‘When he fell ill, I took him to XXX [name of hospital], where they tried to treat him for about three weeks without any improvement. Later, they sent us to YYY [name of hospital].’ (Axe, female, 42 years)

‘He was admitted for two weeks in our local hospital, then discharged. After two weeks, I sent him back because he was not getting better. They gave us more medicines and sent us back home. Those medicines also did not work. I took him back, and only then did the doctor write us a referral letter to bring him to XXX [name of hospital]. They did not refer him on time.’ (Adeh, female, 40 years)

#### Mismanagement of disease at the local health facility

This subtheme indicates how mismanagement of the illness at local health facilities contributed to the delay in primary caregivers presenting children with CKD for medical care. The following statements from three participants show how mismanagement of the disease by health professionals contributed to the delay in presenting children for medical care:

‘They were just tossing us around and made us pay for expensive medicines to keep him alive, but not to cure the disease completely. Every time we went to the health centre and told them about his symptoms, like not passing enough urine, they did not believe me [us] because they could not see any major signs, and said [the] child was okay. This made it difficult for me because my concerns and complaints were not addressed early, making us stay home for almost a year. The disease worsened, so I sent him to another hospital. Then the doctor gave me a referral letter to YYY (name of hospital).’ (Afe, female, 38 years)

‘How can you send a sick child to the hospital and the so-called doctors cannot cure the child? (Very angry tone).’ (Adeh, female, 40 years)

‘Madam, even the district doctors [physician assistants] don’t know anything about this disease. They used my daughter for trials (Nodding the head).’ (Sheh, male, 55 years)

#### Incorrect advice by neighbours

Data revealed that primary caregivers sought advice from their neighbours in their communities prior to making a decision to seek medical care. Some of the advice from neighbours contributed to the delay in presenting the children for medical care as the extracts below from the data demonstrate:

‘My daughter is a fat person, so when we noticed the swelling around the eyes, some neighbours claimed it was haemorrhoids and advised us to use traditional medicine to flush [out] poisons and fat. We did for a while, but there was no improvement.’ (Ace, female, 39 years)

‘Other neighbours told us that the swelling was from worms, and advised us to get some local traditional medicine to apply on the abdomen. We did this, but it didn’t cure him.’ (Adeh, female, 40 years)

### Infrastructural factors contributing to delay in presenting children with chronic kidney disease for medical care

This subtheme emerged from additional factors in the community that are described by primary caregivers as contributing to delays in presenting their children with CKD for medical care. These contributing factors include distance, poor roads and lack of transport to the referral hospital, as the extracts below from two female participants demonstrate:

‘We waited for more than a week to get transport because of the bad road following the rains. It was difficult seeing my child’s condition deteriorating, but I could not do anything because of the bad road.’ (Ace, female, 39 years)

‘The long distance and lack of direct transport was very discouraging at first. It was only when we saw that he was dying in front of our eyes that we decided to brave the distance. We had to set off from home around 4 a.m., but only got here around 11 a.m. the first time we came. The road became very bad during the rainy season.’ (Ahe, female, 44 years)

## Discussion of findings and recommendations

Personal, cultural and spiritual beliefs still play a major role in decision-making regarding seeking Western medical care for chronic illnesses in Ghana and other African countries.^20,21^ For instance, in this study, it was found that primary caregivers’ beliefs in the use of traditional medicines and ancestral and spiritual powers for healing contributed to the delay in presenting their children with CKD to hospital for medical care. This indicates that African people’s personal and cultural beliefs should not be ignored when looking to develop effective strategies to manage and prevent chronic illnesses at primary care level. Although the use of traditional medicines has been found to be effective in treating many ailments in Ghana and other African countries, this practice can also contribute to delays in seeking Western medical care.^22^ Such delays are particularly dangerous in the management of kidney disease in childhood, because failure to diagnose and treat childhood kidney disease early carries a risk for progression into adulthood kidney disease later in life.^1^

Previous studies have identified among primary caregivers two factors, financial constraints and limited knowledge that contribute to delay in seeking medical care in Ghana.^23,24^ Financial constraints affect access to health care for poor people in resource-constrained countries because of their inability to pay for health services.^25^ This indicates a need for consideration of the socio-economic status of communities and the related financial constraints when developing primary prevention strategies for CKD for resource-constrained countries. Failure to recognise this can lead to poor acceptance of primary prevention strategies by communities who cannot access them.

The family serves as a natural support system in planning effective health care for its members.^26^ This probably explains why poor family support was identified as a factor that contributes to delay in presenting children with CKD for health care in Ghana. In this study, poor family support was further complicated by parental marital conflict and a general disrespect for the views of mothers on making decisions to seek timely medical help for their children. Patriarchy remains dominant in Ghana, and thus, some of the cultural practices render women unable to defend their rights and those of their children.^27^ Therefore, developing prevention interventions that fail to acknowledge the effects of patriarchy on women’s decision-making regarding child health is inappropriate and irrelevant.

Two other contributing factors that were identified to delay primary caregivers in presenting their children with CKD for medical care are poor road networks and the bad road conditions during the rainy season. Poor road network is one of the main characteristics of the rural geography of Ghana and is more pronounced in the northern parts of the country where this study was conducted.^28^ This is further complicated by the poor road conditions during the rainy season. This indicates that a multisectoral response is needed in the development of prevention of strategies for CKD and other diseases affecting rural communities in resource-constrained countries.

In acknowledging the influence of traditional and spiritual healers and the use of traditional medicine in Ghana, we also recommend the development of policies that promote a cross-referral system between the Western health care system and traditional and spiritual healers for comprehensive prevention of CKD.

### Strengths and limitations

Although limited to Ghana, the findings of this qualitative study shed some light on the factors that contribute to delays in presenting children with CKD for medical care. These findings urge primary health care practitioners to hasten referrals and provide appropriate health education to primary caregivers about the importance of sending their children for appropriate treatment to prevent the progression of childhood kidney diseases to CKD in later life. The findings may not be generalised to other similar resource-constrained countries. However, the findings provide a basis for researchers in both Ghana and other countries with similar conditions to repeat the study using bigger sample sizes and quantitative methods.

A limitation of the study is the possibility that eliciting the views of the primary caregivers who had delayed presenting their children for medical care could have been perceived as blaming them for the delays. Perceptions of being blamed have a tendency to produce defensive responses or responses attributing delays to other factors other than personal factors. A quantitative study, with equal variables in all levels of the ecological framework, could address this limitation.

## Conclusion

The factors that contribute to primary caregiver delay in presenting children with CKD for medical care in Ghana are mostly beyond the individual primary caregiver’s personal control and include interpersonal, community and infrastructural level factors. This highlights the need for the development of multisectoral and multi-factorial strategies to prevent delays in presenting children with CKD for appropriate medical care. The findings also highlight the importance of considering the personal beliefs and societal norms, such as patriarchy, which still prevails in some communities and affects women’s decision-making on child health, when developing primary prevention interventions for chronic diseases affecting children.

## References

[1] Solarin AU, Madise-Wobo AD, Awobusoyi O, et al Screening for kidney disease in children on World Kidney Day in Lagos State, Nigeria. Afr J Nephrol. 2018;21(1):28–33. 10.21804/21-1-2760

[2] Nasri H, Rafieian-Kopaei M On the occasion of world kidney day 2016; work together to better protect the kidney. J Nephropathol. 2016;5(1):15–18. 10.15171/jnp.2016.03 27047805 PMC4790182

[3] Levey AS, Coresh J Chronic kidney disease. Lancet. 2012(9811);379:165–180. 10.1016/S0140-6736(11)60178-5 21840587

[4] Harambat J, Van Stralen KJ, Kim JJ, Tizard EJ Epidemiology of chronic kidney disease in children. Pediatr Nephrol. 2012;27(3):363–373. 10.1007/s00467-011-1939-1 21713524 PMC3264851

[5] Al-Taiar A, Clark A, Longenecker CJ, Whitty JMC Physical accessibility and utilization of health services in Yemen. Int J Health Geogr. 2010;9:38 10.1186/1476-072X-9-38 20663146 PMC2914054

[6] Lowth M Chronic kidney disease – An update. Pract Nurse. 2013;43(1):34–39.

[7] Mathew T, Corso O Early detection of chronic kidney disease in Australia. Asian Pacific Soc Nephrol. 2009;14:367–373. 10.1111/j.1440-1797.2009.01113.x 19563377

[8] Jayaraman R, Van der Voort J Principles of management of chronic kidney disease. Paediatr Child Health. 2010;20(6):291–296. 10.1016/j.paed.2010.03.016

[9] Crockell YJ Management of chronic kidney disease: An emphasis on delaying disease progression and treatment options. Formulary. 2012;47:228–237.

[10] Wilson C, Campbell MS, Luker AK, Caress AL Referral and management options for patients with chronic kidney disease: Perspectives of patients, generalists and specialists. Health Expect. 2012;18(3):325–334. 10.1111/hex.12025 23216832 PMC5060784

[11] James MT, Hemmelgarn BR, Tonelli M Early recognition and prevention of chronic kidney disease. Lancet. 2010;375(9722):1296–1309. 10.1016/S0140-6736(09)62004-3 20382326

[12] Etheredge H, Paget G Ethics and rationing access to dialysis in resource-limited settings: The consequences of refusing a renal transplant in the South African state sector. Dev World Bioeth. 2015;15(3):233–240. 10.1111/dewb.12067 24953161

[13] Komfo Anokye Teaching Hospital, Kumasi, Ghana 2017 Paediatric renal unit records for 2016. Unpublished.

[14] Duma SE, Khanyile TD, Daniels F Managing ethical issues in sexual violence research using a pilot study. Curationis. 2009;32(1):52–58. 10.4102/curationis.v32i1.879 20225753

[15] Streubert Spezaile HJ. Designing data generation and management strategies In: Streubert Speziale HJ, Carpenter DR, editors Qualitative research in nursing: Advanced in humanistic imperative. 4th ed. Philadelphia, PA: Lippincott Williams & Wilkins, 2007; p. 35–56.

[16] Polit DF, Beck CT Essentials of nursing research: Appraisal evidence for nursing practice. 8th ed. Philadelphia, PA: Lippincott Williams and Wilkins, 2014; 309 p.

[17] Schneider MJ Introduction to public health. 5th ed. Burlington: Jones & Bartlett Learning Publications, 2017; p. 211.

[18] Aziato L The dynamics of female education from the basic to the tertiary levels in Ghana: Challenges and reflections. Social Work Practitioner-Researcher. 2016;28(3):330–343. 10.25159/2415-5829/1843

[19] Appiah M, Blay D, Damnyag L, Dwomoh KF, Pappinen A, Luukkanen O Dependence on forest resources and tropical deforestation in Ghana. Environ Dev Sustain. 2009;11(3):471–487. 10.1007/s10668-007-9125-0

[20] Gyasi RM, Asante F, Yeboah YJ, Abass K, Mensah CM, Siaw LP Pulled in or pushed out? Understanding the complexities of health beliefs and motivations for traditional medicine utilisation in Ghana. Qual Prim Care. 2015;23(4):249–258.

[21] Kretchy IA, Owusu-Daaku F, Danquah D Spiritual and religious beliefs: Do they matter in the medication adherence behaviour of hypertensive patients? Bio Psycho Soc Med. 2013;7:15 10.1186/1751-0759-7-15 PMC3854617 24138844

[22] Gyasi RM, Mensah CM, Siaw LP Predictors of traditional medicines utilisation in the Ghanaian health care practice: Interrogating the Ashanti situation. J Commun Health. 2015;40(2):314–325. 10.1007/s10900-014-9937-4 25173694

[23] Asoogo C, Duma S Factors contributing to late breast cancer presentation for health care amongst women in Kumasi, Ghana. Curationis. 2015;38(1):1287 10.4102/curationis.v38i1.1287 PMC6091661 26841912

[24] Roomizadeh P, Taheri D, Abedini A, et al Limited knowledge of chronic kidney disease and its main risk factors among Iranian community: An appeal for promoting national public health education programs. Int J Health Policy Manag. 2014;2(4):161–166. 10.15171/ijhpm.2014.37 24847481 PMC4025092

[25] Macha J, Harris B, Garshong B, et al Factors influencing the burden of health care financing and the distribution of health care benefits in Ghana, Tanzania and South Africa. Health Policy Plan. 2012;27(Supl 1):i46–i54. 10.1093/heapol/czs024 22388500

[26] Burns EC, Dunn MA Introduction to functional health patterns and health promotion In Burns CE, Dunn AM, Brady MA, Star NB, Blosser CG, editors Pediatric primary care. 5th ed. Philadelphia, PA: Saunders-Elsevier Inc; 2013 p. 138–148.

[27] Tenkorang EY, Owusu AY, Yeboah EH, Bannerman R Factors Influencing domestic and marital violence against women in Ghana. J Fam Violence. 2013;28(8):771–781. 10.1007/s10896-013-9543-8

[28] Jha V, Garcia-Garcia G, Iseki K, Li Z, et al Chronic kidney disease: Global dimension and perspectives. Lancet. 2013;382(9888):260–272. 10.1016/S0140-6736(13)60687-X 23727169

[29] Atuoye NK, Dixon J, Rishworth A, et al Can she make it? Transportation barriers to accessing maternal and child health care services in rural Ghana. BMC Health Serv Res. 2015;15:333 10.1186/s12913-015-1005-y 26290436 PMC4545969

